# A history of FMD research and control programmes in Southeast Asia: lessons from the past informing the future

**DOI:** 10.1017/S0950268819000578

**Published:** 2019-04-05

**Authors:** Stuart D. Blacksell, Jarunee Siengsanan-Lamont, Somjai Kamolsiripichaiporn, Laurence J. Gleeson, Peter A. Windsor

**Affiliations:** 1 Mahidol Oxford Tropical Medicine Research Unit, Faculty of Tropical Medicine, Mahidol University, Bangkok, Thailand; 2 Nuffield Department of Medicine, Centre for Tropical Medicine and Global Health, John Radcliffe Hospital, Oxford, UK; 3 Independent Veterinary Consultant, Canning Vale, Western Australia, Australia; 4 Independent Veterinary Consultant, Bangkok, Thailand; 5 Independent Veterinary Consultant, Killarney, Victoria, Australia; 6 Sydney School of Veterinary Science, University of Sydney, Camden, NSW, Australia

**Keywords:** Buffalo, cattle, foot and mouth disease virus, pig, Southeast Asia

## Abstract

Foot and mouth disease (FMD) is a major animal health problem within Southeast Asia (SEA). Although Indonesia and more recently the Philippines have achieved freedom from FMD, the disease remains endemic on continental SEA. Control of FMD within SEA would increase access to markets in more developed economies and reduce lost productivity in smallholder and emerging commercial farmer settings. However, despite many years of vaccination by individual countries, numerous factors have prevented the successful control of FMD within the region, including unregulated ‘informal’ transboundary movement of livestock and their products, difficulties implementing vaccination programmes, emergence of new virus topotypes and lineages, low-level technical capacity and biosecurity at national levels, limited farmer knowledge on FMD disease recognition, failure of timely outbreak reporting and response, and limitations in national and international FMD control programmes. This paper examines the published research of FMD in the SEA region, reviewing the history, virology, epidemiology and control programmes and identifies future opportunities for FMD research aimed at the eventual eradication of FMD from the region.

## Introduction

Foot and mouth disease (FMD) is endemic in the majority of Southeast Asia (SEA) and remains a major animal health problem within the region [1]. SEA comprises a continental component (Thailand, Peninsular Malaysia, Cambodia, Lao People's Democratic Republic (Lao PDR), Vietnam and Myanmar) and a series of island countries (Philippines, Singapore, Brunei Darussalam and Indonesia) and states (Sabah and Sarawak – Malaysia) and is represented by the geopolitical organisation, Association of Southeast Asian Nations (ASEAN). FMD is endemic on continental SEA, while the island countries and states are FMD-free without vaccination. The region is economically diverse which influences the direction of commerce in many sectors including agriculture and in particular, transboundary trade of livestock and their products. This review examines previously published research to summarise the history of FMD in the ASEAN member states, discussing the virology, epidemiology and control programmes, in addition to identifying future opportunities for FMD control and the eventual eradication of FMD from the region.

## FMD situation in SEA

Historically, FMD has been recognised in SEA for approximately 150 years (Table 1) with early outbreaks recorded in Indonesia, Malaysia and the Philippines. Serotype O is dominant throughout SEA, although serotypes A and Asia 1 have caused outbreaks in most countries in the region with the exception of Indonesia. Serotype C was confined to historical outbreaks in the Philippines between 1976 and 1994. Singapore has only reported a serotype A outbreak in 1973 and Brunei Darussalam has never reported an FMD outbreak.

**Table 1.** Summary of historical and contemporary FMD outbreaks in SEA as reported on the WRLFMD website (http://www.wrlfmd.org)

Country	FMD-free	O	A	Asia 1	C	Untyped
Brunei Darussalam	FMD-free without vaccination					
Cambodia	Endemic	1989, 1992, 1994, 1998–2000, 2004–2008, 2010–2013, 2015–2016	2006–2008, 2015–2016	1980–1981, 1988, 1990–1991, 1993–1994, 1997		2006, 2009, 2011
Indonesia	FMD-free without vaccination	1952, 1956–1958, 1962 (Bali), 1972–1974, 1983				Sept 1887 (Malang, East Java), 1892 (East Java, Sumatra), 1902 (Sulawesi), 1906 (Kalimantan, Madura Island), 1907 (Sulawesi), 1911 (West Nusa Tenggara), 1913 (Madura Island)
Lao PDR	Endemic	1978, 1981–1982, 1984, 1987–1990, 1993, 1998–2001, 2003–2013, 2016–2017	2003, 2006–2008, 2014–2015	1984, 1991–1993, 1996, 1998		
Malaysia	Endemic	1978–1981, 1983–1984, 1992, 1994–1996, 1999, 2000–2016	1973, 1995, 1997, 2002–2005, 2007–2014	1985–1986, 1999		1860s, 1909 (Kedah), 1910 (Penang), 1917 and 1929 (Pahang), 1936 (Perak and Selangor), 1938 (Perak)
Myanmar	Endemic	1956–1958, 1971, 1977–1978, 1982, 1989, 1996, 1998–2011, 2015–2017	1971, 1978, 2010, 2015	1958, 1971, 1977–1978, 1982, 1989, 1991, 1997, 2000–2001, 2005, 2017		
Philippines	FMD-free without vaccination	1954, 1958–1959, 1965–1968, 1972–1975, 1984?, 1988–1991, 1994–2005	1941?, 1975–1981		1976–1981, 1983–1990, 1994	30 June 1902, 1920, enzootic between 1930 and 1939
Singapore			1973			
Thailand	Endemic	1958, 1960, 1980, 1991–1992, 1994–1995, 1999–2017	1953–1957, 1960, 1973, 1986–1988, 1991–1993, 1997–2017	1954–1958, 1960, 1985, 1987?, 1990–1991, 1994–1996, 1998		
Vietnam	Endemic	1956, 1967, 1969, 1997, 1999–2018	2004–2007, 2009–2010, 2012–2017	1992, 2005–2007		

?, as reported on the WRLFMD website.

**Fig. 1.**
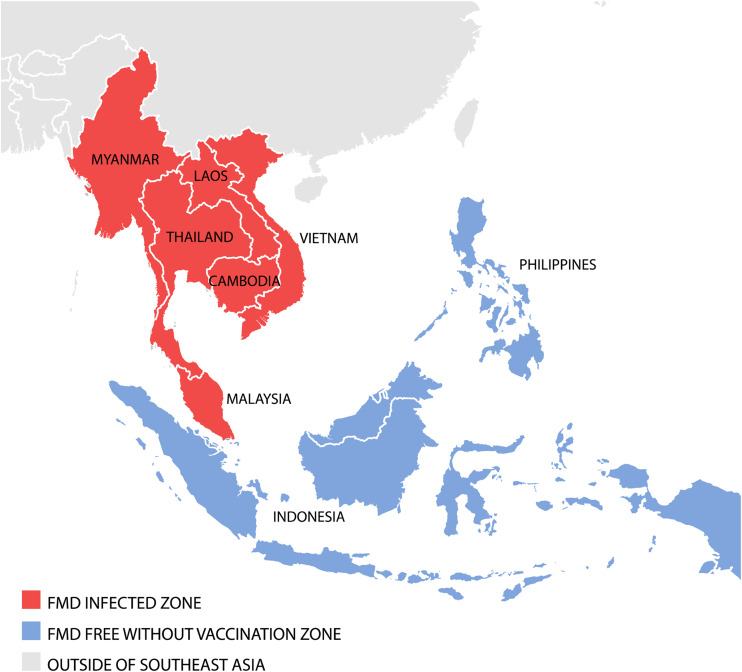
Map of SEA indicating FMD disease status.

As of 2018, FMD is endemic throughout continental SEA. Singapore, Brunei Darussalam, Indonesia and Philippines are recognised as FMD-free without vaccination (see Fig. 1). Over the past decade (2007–2017), 4961 FMD outbreaks from Cambodia, Lao PDR, Malaysia, Myanmar, Thailand and Vietnam have been reported to the ASEAN Regional Animal Health Information System (ARAHIS) and the Office International Epizooties (OIE) World Animal Health Information System online notification application (WAHIS) (Table 2). For the first 5-year reporting period, outbreaks gradually increased approximately fourfold from 307 (2007) to 1214 (2011). This rise could be partly attributed to improved awareness of the importance of, and the need for reporting, although it is likely there was a contribution from increased transboundary trade in livestock and their products due to an escalation in regional demand for meat as national economies developed [2]. Since 2011, when a major epidemic of serotype O (mainly topotype Mya-98) emerged in the region and spread to PR China, Korea and Japan [3], the number of reported outbreaks declined then rebounded to approximately 400 outbreaks a year in 2015. Overall, of the 4961 FMD outbreaks, 1773 (35.6%) outbreaks had an FMD serotyping result of which the majority were serotype O (77.9%; 1416 outbreaks), followed by serotype A (19.8%; 351 outbreaks) and serotype Asia 1 (0.3%; 6 outbreaks). Interestingly, 3143 outbreaks (63.4%) had no serotyping result.

**Table 2.** Summary of FMD outbreaks reported to ARAHIS (2007–2011)/RAHIS (2012–2017) from 2007 to June 2017

Country	Serotype	2007	2008	2009	2010	2011	2012	2013	2014	2015	2016	2017	Total
Cambodia	O	0	2	0	18	0	20	50	13	1	1	0	**105**
	A	0	2	0	0	0	0	0	1	0	2	1	**6**
	Asia 1	0	0	0	0	0	0	0	0	2	0	0	**2**
	Not serotyped	6	58	65	206	124	0	30	75	52	90	24	**730**
Lao PDR	O	8	16	11	0	0	0	0	0	3	0	0	**38**
	A	0	2	0	0	0	0	1	0	7	0	0	**10**
	Asia 1	0	0	0	0	0	0	0	0	0	0	0	**0**
	Not serotyped	122	87	28	0	0	0	6	1	0	0	0	**244**
Malaysia	O	69	126	17	7	2	48	11	8	9	60	0	**357**
	A	1	2	2	0	4	0	0	0	0	3	0	**12**
	Asia 1	0	0	0	0	0	0	0	0	0	0	0	**0**
	Not serotyped	0	0	80	58	13	50	13	0	4	21	0	**239**
Myanmar	O	14	7	13	3	3	1	9	0	15	9	4	**78**
	A	0	0	0	1	0	0	0	1	13	0	0	**15**
	Asia 1	0	0	0	0	0	0	0	0	0	0	1	**1**
	Not serotyped	0	4	10	6	1	2	0	1	2	2	1	**29**
Thailand	O	29	40	28	19	5	9	4	36	69	94	0	**333**
	A	9	22	12	2	5	10	41	54	27	52	0	**234**
	Asia 1	0	0	0	0	0	-	0	0	0	0	0	**0**
	Not serotyped	4	10	25	13	8	7	8	61	131	94	0	**361**
Vietnam	O	30	61	112	161	8	34	19	39	22	19	0	**505**
	A	0	0	32	0	0	-	6	18	17	11	0	**84**
	Asia 1	3	0	0	0	0	0	0	0	0	0	0	**3**
	Not serotyped	0	0	156	230	1043	0	23	20	31	37	0	**1540**
**Sub-totals**	O	150	252	181	208	18	112	93	96	119	183	4	**1416**
	A	10	28	46	3	9	0	48	74	64	68	1	**361**
	Asia 1	3	0	0	0	0	0	0	0	2	0	1	**6**
	Not serotyped	132	159	364	513	1189	59	80	158	220	244	25	**3143**
**Total**		**307**	**464**	**591**	**724**	**1214**	**181**	**221**	**328**	**405**	**495**	**31**	**4961**

## Characteristics and chronology of FMD viruses causing disease in SEA

### Serotype O

Of the 10 serotype O topotypes recognised worldwide, Europe–South America (Euro-SA), Middle East–South Asia (ME-SA), SEA, Cathay (CHY), West Africa (WA), East Africa 1 (EA-1), East Africa 2 (EA-2), East Africa 3 (EA-3), Indonesia-1 (ISA-1) and Indonesia-2 (ISA-2), three distinct topotypes are recognised in SEA: O/SEA, O/ME-SA and the O/CHY [4–7]. The Indonesian topotypes O/ISA-1 and O/ISA-2 isolated from outbreaks in 1962–1983 and 1972–1974, respectively [8], are now considered extinct [7].

The O/SEA topotype is characterised by two distinct lineages, O/SEA/Mya-98 and O/SEA/Cam-94 of which the former lineage is dominant [1, 4], and genetic studies suggest that these lineages arose from a common ancestor approximately 35 years ago [4]. Viruses belonging to the O/SEA/Mya-98 lineage have been detected in all six countries of continental SEA over the past 15 years, while the O/SEA/Cam-94 lineage was only identified between 1989 and 2003 and may be considered extinct [4].

Towards the end of 1999, the O/ME-SA/PanAsia lineage was introduced into continental SEA, and by April 2000, all countries had experienced outbreaks [6]. The O/ME-SA/PanAsia lineage is divided into PanAsia and PanAsia-2 with the latter only been reported in SEA in Malaysia in 2003, 2005 and 2009 [9, 10]. More recently, the O/ME-SA/Ind-2001 lineage that was indigenous in the Indian subcontinent (12) caused outbreaks in North Africa, the Middle East, SEA, the Far East and the FMD-free islands of Mauritius between 2013 and 2017 [11, 12]. It first appeared in SEA causing outbreaks in Lao PDR and Vietnam in 2015 and Myanmar in 2016 [11–15]. The O/ME-SA/Ind-2001 lineage has demonstrated sequence heterogeneity with two co-evolving divergent sub-lineages (Ind-2001d and Ind-2001e) [12], which have both caused outbreaks in SEA. The O/ME-SA/Ind-2001e lineage was introduced into Myanmar during 2017, which subsequently spread further to Thailand, Vietnam and Malaysia [12, 16] (Table 3).

**Table 3.** Summary of FMD serotypes, topotypes and lineages (in parentheses). Data based as reported on the WRLFMD website (http://www.wrlfmd.org) and publications

Country	Serotype	2010	2011	2012	2013	2014	2015	2016	2017	2018
Cambodia	O	ME-SA (PA^a^)	ME-SA (PA)	ME-SA (PA)	ME-SA (PA)		ME-SA (PA)		ME-SA (PA)	
	A						ASIA (Sea-97)		ASIA (Sea-97)	
Lao PDR	O	ME-SA^b^ (PA) SEA^c^ (Mya^d^-98)	ME-SA (PA)	ME-SA (PA)	ME-SA (PA)		ME-SA (Ind^e^-2001d)	ME-SA (PA)	ME-SA (PA)	
	A	ASIA				ASIA (Sea-97)	ASIA (Sea-97)			
Malaysia	O	SEA (Mya-98)	SEA (Mya-98)	SEA (Mya-98)	SEA (Mya-98)	SEA (Mya-98)	SEA (Mya-98)	SEA (Mya-98)	ME-SA (Ind-2001e)	ME-SA (Ind-2001e)
	A		ASIA (Sea-97)	ASIA (Sea-97)	ASIA (Sea-97)	ASIA (Sea-97)				
Myanmar	O	SEA (Mya-98)					SEA (Mya-98)	ME-SA (Ind-2001d)	ME-SA(Ind-2001d) ME-SA(Ind-2001e) SEA (Mya-98)	
	A	ASIA					ASIA (Sea-97)			
	Asia 1								ASIA(G-VIII)	
Thailand	O	SEA (Mya-98)	ME-SA (PA) SEA (Mya-98)	ME-SA (PA) SEA (Mya-98) CHY	SEA (Mya-98)	SEA (Mya-98)	ME-SA (PA) SEA (Mya-98)	ME-SA (PA) SEA (Mya-98)	ME-SA (PA) ME-SA (Ind-2001e) SEA (Mya-98)	ME-SA (PA) ME-SA (Ind-2001d)
	A	ASIA (Sea-97)	ASIA (Sea-97)	ASIA (Sea-97)	ASIA (Sea-97)	ASIA (Sea-97)	ASIA (Sea-97)	ASIA (Sea-97)	ASIA (Sea-97)	ASIA (Sea-97)
Vietnam	O	ME-SA (PA) SEA (Mya-98)	ME-SA (PA) SEA (Mya-98)	ME-SA (PA)	ME-SA (PA)	ME-SA (PA) SEA (Mya-98)	ME-SA (PA) ME-SA (Ind-2001d) SEA (Mya-98)	CHY ME-SA (PA) SEA (Mya-98)	CHY ME-SA (PA) ME-SA (Ind-2001d) ME-SA (Ind-2001e) SEA (Mya-98)	
	A	ASIA (Sea-97)	ASIA (Sea-97)	ASIA (Sea-97)	ASIA (Sea-97)	ASIA (Sea-97)	ASIA (Sea-97)	ASIA (Sea-97)	ASIA (Sea-97)	

a PA, PanAsia.

b ME-SA, Middle East–South Asia.

c SEA, Southeast Asia.

d Mya, Myanmar.

e Ind, India.

The emergence of the pig-adapted strain O/CHY topotype was attributed to the movement of pigs across the Chinese border into northern Vietnam in 1997, and presumed onto Luzon Island of the Philippines in 1994 by pig products as ‘swill’ via Manila airport [2, 17], with limited spread into Thailand and Malaysia in 2005 [18]. The O/CHY topotype was later detected in Thailand in 2012 and Vietnam in 2015–17 [11] (Table 3). It remains uncertain whether this virus is endemic in SEA or is occasionally reintroduced through movement of animal or products.

### Serotype A

Historical antigenic studies during the 1960s with global serotype A viruses discriminated 32 subtypes (lineages) [19]. Subsequent studies of FMD serotype A viruses of Asian origin during the 1980s found that lineage A_22_ (Isolate A22/IRQ/24/64) was dominant [20] with lineage A_15_ (Isolate A_15_/Bangkok/TAI/60) also causing disease [21]. More recently, comparison of approximately 300 VP1 sequences of serotype A viruses demonstrated three major geographically-restricted topotypes: (i) Euro-SA, (ii) Asia and (iii) Africa [7]. The Asian topotype comprises the A_15_, A_22_, Iran-05, Thai-87, Sea-97 and G-VII lineages and all the serotype A viruses found in SEA belong to the Asian topotype (Table 3) [1, 4, 6, 22, 23]. Closely related serotype A viruses have circulated within and between SEA countries. For example, samples from outbreaks in Malaysia in 2002 (A/MAY/2/2002) and 2009 (A/MAY/9/2009) grouped with isolates collected in the same years from Thailand and Vietnam, respectively [4]. Serotype A virus from outbreaks in Lao PDR in 2003 showed 99.84% identity with the Malaysian A/MAY/4/2003 isolate indicating a common origin [23]. Cambodian isolates from 2006 and 2008 and those collected between 2008 and 2010 from Thailand, and in 2009 in Malaysia, grouped with contemporary viruses from Vietnam [13]. Myanmar first detected serotype A virus in 2015 from buffalo samples collected in 2008/2009 and this virus was closely related to those detected later in Thailand in 2014/2015 [11]. These data on virus relationships are good evidence of the way that the virus moves around the region, most likely with animal trading.

### Serotype Asia 1

Asia 1 viruses have three antigenic subtypes [7] and a genetic study of 44 serotype Asia 1 strains from Bangladesh, Bahrain, Bhutan, Burma, Cambodia, Greece, Hong Kong, India, Israel, Kuwait, Lebanon, Malaysia, Nepal, Oman, Pakistan, Saudi Arabia, Tajikistan, Thailand, Turkey and Yemen collected between 1954 and 1992 demonstrated that all isolates could be included in a single topotype [22]. A study examined Asia 1 viruses responsible for outbreaks in Asia from 2003 to 2007 and classified them into six groups based on VP1 sequence [24]. Viruses in group IV belonged to a larger, more diverse, group of viruses that were found only in SEA and Hong Kong from 1974 through 2006, and interestingly, only two viruses originating from SEA fell outside this supergroup, Bangkok/Thailand/60 (an old Thai vaccine virus strain) and ASIA1/MYA/2/2001 [24]. Within group IV, Asia 1 viruses that caused outbreaks in Yunnan Province of PR China (that borders Lao PDR, Myanmar and Vietnam) and Vietnam in 2005 and 2006 were related to viruses from Thailand in 1998 and Myanmar in 2005 [24]. A separate study further confirmed and delineated outbreaks (homology <95%) that occurred in Myanmar in 2005 and 2006 [18]. The 2005 Myanmar outbreaks were closely related to the Asia 1 virus reported in PR China during 2005, whilst the 2006 outbreaks were more closely related to the outbreaks in Vietnam in 2005–2006 [18]. No other evidence of this serotype was found elsewhere [11] until an outbreak in Rakhine State, Myanmar in 2017 (Table 3), where sequencing data demonstrated that the outbreak was caused by a new introduction of the G-VIII lineage from Bangladesh [25].

### Serotype C

Serotype C viruses isolated in Europe and South America were originally classified into five antigenic subtypes, C1–C5 [26]. Comparisons of partial VP1 sequences classified FMD serotype C viruses into three topotypes: Euro-SA, Africa and Asia [6]. Serotype C was introduced into the Philippines in 1976 (C-Philippines) and this virus was very closely related to the South American vaccine strain, C3/Resende/Brazil/55 [6]. No outbreaks of serotype C have been recorded worldwide for the past 14 years [27].

## Risk factors that influence the spread of FMD in SEA

### Transboundary livestock movement and poor biosecurity

Livestock movement and trade in livestock products are the greatest risk factor in the transboundary spread of FMD in SEA involving complex and rapidly changing market chains linking producers to consumers. A deep understanding of these market chains is essential to understanding movements and pathways of FMD spread. Continental SEA has relatively open borders, and as such, regional trade routes in large ruminants and pigs are well established although the volume and direction of animal movement can be quite variable. The supply and demand for animals and animal products fluctuate considerably within these countries from year to year particularly around the times of national festivals [9]. A number of excellent reviews have examined the role of animal movement in the spread of FMD in Asia and other regions [1, 9, 28]. Countries such as Myanmar, Cambodia and Lao PDR tend to be thoroughfares for livestock movement on their way to higher value markets in Thailand, Malaysia, Vietnam [9] and particularly PR China in recent years with anecdotal reports that over 1 million cattle are now entering southern PR China annually. Studies have identified critical points for transboundary animal disease amplification and transmission, such as holding facilities and livestock markets that have high concentrations of animals that provide opportunities for extensive mixing of livestock from different origins that are destined for different locations [28]. Due to the extensive shared borders, large-scale unofficial cross-border movement, a lack of effective biosecurity and variable and often low levels of compliance with animal health regulations, regional cooperation is paramount if countries are to control transboundary animal diseases such as FMD [9, 28].

### Host-related factors

The capability of some species to become FMD carriers may be an important factor in the spread of FMD [29]. It is well established that both vaccinated and unvaccinated cattle (*Bos taurus*, *Bos indicus*) can be persistently infected with FMD for many months [29] but the role of the Asian domestic water buffalo (*Bubalus bubalis*) as an FMD carrier that can maintain FMD persistence in the absence of clinical disease was only recently confirmed [30]. Interestingly, studies in Vietnam have demonstrated that persistently infected animals do not transmit the disease animals to naive animals [31]. In SEA, pigs have been reported as both refractory [32] and highly susceptible [5] to FMD infection, illustrating the important role of adaption and species susceptibility to different FMD strains such as the pig-adapted O/CHY topotype [33]. The role of small ruminants in the epidemiology of FMD in SEA is unclear which highlights the need for further studies to understand the function of all species for disease control or eradication.

### Vaccine-related issues

FMD vaccines used in SEA include both imported and locally produced sources. Significant regional vaccine production capabilities are available, and since the 1960s, the Thai Government's Department of Livestock Development (DLD), Bureau of Veterinary Biologics Pak Chong facility has produced FMD vaccines based on representative contemporary Thai field strains. Myanmar also has its own vaccine production facility that manufactures for local use, although it is unclear what serotypes and strains are employed. Historically, Thai FMD vaccine production commenced in 1960, using serotype A, O and Asia 1 viruses isolated in the Bangkok area that were designated O/BKK/60, A/BKK/60 and Asia1/BKK/60. Subsequently, Thai vaccine strains designated O/Udonthani/1987, Asia1/Petchburi/1985, A/Nakhonpathom/1987, A/Sakolnakorn/1997, A/Lopburi/2012 and A/Saraburi/1987 have been used. The contemporary Thai strains used for FMD control in Thailand include O/Udonthani/1987, Asia1/Petchburi/1985, A/Sakolnakorn/1997 and A/Lopburi/2012. Vaccines imported to SEA include O/3039, O_1_/Manisa, A_22_/Iraq, A/IRN/05 and Asia1/Shamir vaccine strains.

There is an increasing need to address the difficulties of achieving sufficient herd immunity in vaccination programmes and to continually monitor the efficacy and potency of vaccines in the field to ensure their effectiveness to control FMD driven by increasing livestock trade in the region [2, 34]. A limited number of published studies have examined the efficacy of locally produced and imported vaccines against viruses causing disease in SEA. Studies in the late 1970s and 1980s determined that serotype A and Asia 1 viruses from Thailand were found to be divergent requiring the development of new vaccines [21, 35, 36], whilst serotype O isolates were found to be largely homologous [37, 38] and vaccines of the time provided good protection. However, the evolution and spread of O/ME-SA/Ind-2001 lineage viruses to SEA has raised question regarding vaccine efficacy of the conventional complement of vaccine strains mainly O/3039 and O_1_/Manisa. A recent study has demonstrated the effectiveness of high potency (⩾6PD_50_/dose) O_1_/Manisa strain vaccine [39] against O/ME-SA/Ind-2001 lineage viruses. Another recent study has tested a current Indian vaccine strain O/IND/R2/75 against 23 Indian field isolates and 19 field isolates of ME-SA topotype mainly from Asia and Africa. It revealed a good match to 79% of the viruses indicating that the vaccine strain is broadly cross-reactive and could be used to control FMD in other countries [40]. A later and more extensive study with a wider range of vaccines (O/HKN/6/83, O/IND/R2/75, O/SKR/2010 and O/PanAsia-2 and one putative O/MYA/2009) and viruses from SEA also demonstrated acceptable levels of neutralisation *in vitro* indicating that these could be possible candidates for future vaccine strains [41].

## FMD eradication and control programmes in SEA

### The benefits of FMD control or eradication

There is a financial imperative to control or eradicate FMD. Previous studies generated a benefit-cost ratio (BCR) which is the ratio of the benefits of a project or proposal, expressed in monetary terms, relative to its costs. Benefit-cost studies at a national or SEA regional level have demonstrated the positive financial benefits of FMD control or eradication. Many of the early studies assumed increased access to export markets following FMD eradication with BCR of 15:1 (Thailand) [42] and 12:1 (Philippines) [43]. However, assuming no additional export markets following FMD eradication, the BCR has been reported between 1.08:1 (Thailand) [44], 1.6:1 (Philippines) [43], 3:1 (SEA) [45, 46], 3.7:1(Thailand) [42] and 11.8:1 (Thailand) [47]. The disparity in the BCR results highlights the need for additional studies using contemporary assumptions to better understand the benefits of FMD control or eradication in a financial and social context.

Production losses associated with FMD outbreaks have a large impact on the world's poorest farming communities, where more people are dependent on livestock for their livelihoods. As well as visible direct effects, the disease reduces herd fertility leading to less efficient herd structures and discourages the use of FMD-susceptible, high productivity breeds [48]. Overall the direct losses limit livestock productivity affecting food security and contribute to rural poverty [48, 49]. A number of recent studies have quantified the socio-economic impacts of FMD and the opportunities and advantages for control of the disease for smallholders in Cambodia [50, 51] and Lao PDR [5, 52–55]. In Cambodia, it was demonstrated that the average post-FMD loss varied from US$216.32, a 54% reduction from the pre-FMD value because of weight loss and treatment costs, to US$370.54, a 92% reduction from pre-FMD values if the animal was treated, died and a rental draft replacement animal was required [56]. In Lao PDR, estimated financial impact of FMD at the village level revealed losses of US$30 881 per village, although this value depended on the number of households affected within the village [57].

### FMD control methods

#### Estimating the benefits of vaccination in smallholder farms

In the FMD endemic countries of SEA, the control of FMD has largely relied on vaccination, as ‘stamping out’ (slaughter) is generally impractical and/or culturally inappropriate for smallholder production systems and compliance with animal movement controls often difficult to achieve.

In SEA, studies have demonstrated evidence that FMD vaccination would reduce the financial impact of FMD on smallholder livelihoods with benefits of small-scale vaccination per smallholder family at US$22–82 [51] in Lao PDR and US$28–51 [58] in Cambodia. In a case study in Lao PDR [53], FMD vaccination substantially reduced estimated financial losses associated with FMD in a fully vaccinated village (US$1.7–1.9 per cow/buffalo) compared with unvaccinated villages (US$52.4–70.8 per cow/buffalo). In Cambodia, a benefit of US$31.48 per animal vaccinated was calculated [56] and it was hypothesised that implementing a 5-year biannual vaccination programme at a cost of US$6.3 an animal per year would give a BCR of 1.40 when assuming there is one major epizootic during the 5-year vaccination programme [59]. Another recent study examined the risk factors associated with FMD outbreaks in Lao PDR that underlined the value of implementing basic on-farm biosecurity using quarantine and improved husbandry measures to minimise FMD circulation at the household level [55]. The study reported that 30.2% (*n* = 19) of households quarantined new livestock for a minimum of 2 weeks prior to introduction to the herd which was a significantly protective factor for reducing clinical FMD [55].

### Successful FMD eradication programmes

#### Indonesia

Indonesia has been recognised as FMD-free without vaccination since 1986. The first reported outbreak of FMD in Indonesia was in East Java in 1887 followed by outbreaks on Madura Island in 1906 and 1913 [60]. FMD became endemic in East Java and spread throughout Java and other islands including Sumatra (1892), Kalimantan (1906), Sulawesi (1902), West Nusa Tinggara (1911) and Bali (1962) [60]; identified as serotype O by World Reference Laboratory for FMD (WRLFMD), Pirbright in 1973 [60]. In 1974, an FMD eradication programme was implemented supported by the Australian International Development Assistance Bureau (AIDAB) [2, 61] providing AU$7.865 million for FMD vaccine, vehicles and vaccinating equipment as well as the services of consultants and advisers for field control of the disease (Anonymous, 1979).

**Fig. 2.**
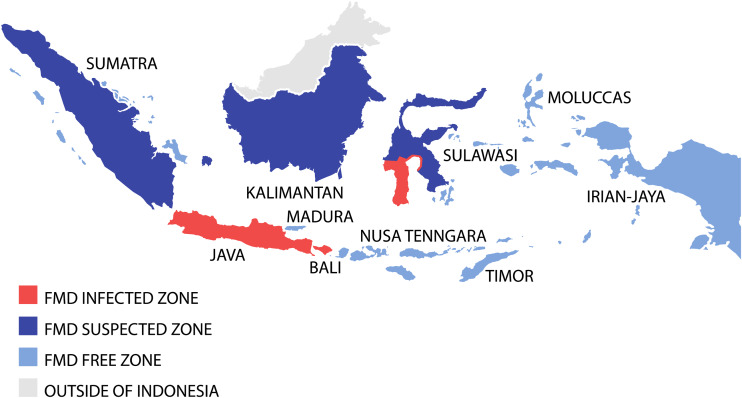
Map demonstrating the progressive vaccination zones use during the Indonesian FMD eradication campaign. Adapted from Soehadji *et al*. [60].

To achieve successful FMD eradication, the Indonesian livestock authorities divided the country into three FMD zones; a disease-free zone (East and West Nusa Tenggara, Irian Jaya (now Western New Guinea or Papua), Moluccas, East Timor (now Timor Leste)); a suspected zone (Kalimantan, Sumatra, Sulawesi); and an infected zone (Java, Bali, South Sulawesi) (see Fig. 2) [60]. Strict animal movement and quarantine were introduced to protect the disease-free zone and routine surveillance was conducted out in the suspected zone. In the infected zone, a mass vaccination programme was carried out using both ‘crash’ and ‘low-speed’ programmes. The ‘crash’ method employed mass vaccination in order to prevent reappearance of the disease in the infected areas, and the ‘low-speed’ method was implemented gradually but in an intensive manner to cover the areas where there was potential for disease to spread to the non-infected areas [60]. The vaccination procedure was similar for both systems and performed annually three times in 3 years, for all livestock more than 3 months old. Pigs were vaccinated only in infected herds, whereas goats and sheep were vaccinated voluntarily because they have been shown experimentally to be possible disease carriers. Vaccination coverage was estimated to involve at least 80% of the livestock population. Vaccinated animals were identified by ear markings and there was a strict control of livestock vehicles to minimise animal movement into the vaccination area. Intensive epidemiological surveillance was performed continuously during the programme to monitor the possible reappearance of cases, and after 3 years, many regions were declared free of FMD.

However, in July 1983, an outbreak of FMD serotype O occurred in Cepu district on the border between Central and East Java [62] and spread over much of the island infecting 13 976 animals. In this situation, control measures included stamping out, control of livestock movements and disinfection of vehicles, closure of infected areas to prevent livestock movement, control at abattoirs including slaughter and meat distribution, control at quarantine stations, isolation and treatment of livestock and disinfection of livestock premises, mass vaccination, reporting of cases, disease surveillance and use of extension services [60]. The last cases of FMD occurred in December 1983 and the final vaccinations were conducted in Java in 1985 leading to declaration of Indonesia as free of FMD in 1986 [2, 60].

#### Philippines

The Philippines is recognised as maintaining FMD-free status without vaccination. FMD was first reported in the Philippines on 30 June 1902 as a result of importation of beef cattle from Hong Kong to Manila [17]. Since the first reported FMD outbreak in 1908, the Philippines experienced sporadic outbreaks until an major epidemic emerged in 1994 which principally affecting pigs due to introduction of the first recognised porcinophillic strain [63]. Samples collected from outbreaks in 1959, 1966, 1972 and 1975 were sent to FMD WRL, Pirbright, UK and identified as serotype O_1_. Serotype A_22_ was identified in 1975 from an outbreak in central Luzon, central Visayas and Cotabato. In February 1976, serotype C_3_ was reported from the central Visayas. Between 1984 and 1986, FMD occurred on Luzon, with sporadic cases in the island provinces of Masbate and South Cotabato during 1986–88. No major outbreaks of FMD were reported from 1989 to 1992 except for a few sporadic cases of which 7% were serotype A, 57% were serotype O and 34% were serotype C [17].

**Fig. 3.**
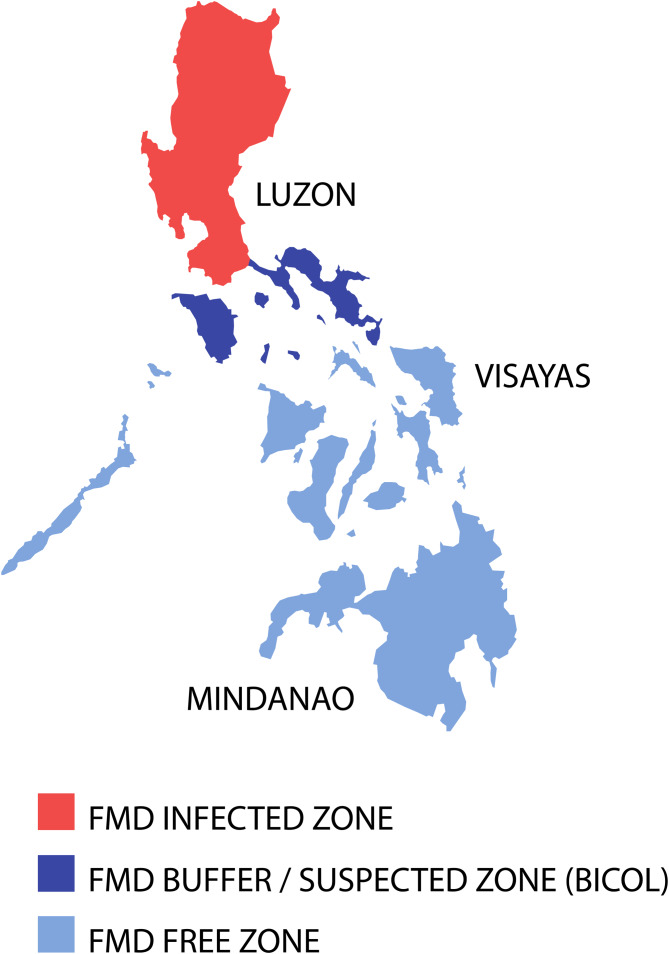
Map of the Philippines displaying the zoning for FMD eradication in 1998. Adapted from Windsor *et al*. [63].

When the epidemic porcinophilic serotype O (CHY topotype) occurred from 1994 through to 2005 in the Philippines [63], a national plan to eradicate FMD supported by Australia and facilitated by FAO was initiated in 1996 [63]. The programme was based on three main components: (1) disease monitoring and surveillance, (2) public awareness and (3) animal movement management, although mass vaccination programmes were included, involving some of the commercial and smallholder piggeries in certain areas also occurred [63]. The national plan used a progressive zoning approach by classifying different regions based on their FMD status [64]. Eradication of the disease commenced from northern and southern Luzon provinces moving towards central Luzon [63]. Importantly, a disease surveillance buffer zone in the southern Luzon region of Bicol (see Fig. 3) was established to protect the Visayas and Mindanao from infection. Following intensive disease control work that included a widespread public awareness ‘school on the air’ programme to communicate through women's groups to provide simple biosecurity messages that encouraged the cooking of swill prior to feeding of pigs in smallholder households, eventual elimination of the disease in Luzon was achieved [63].

Critical pathway analysis identified high-risk areas for FMD spread being livestock markets, holding yards and abattoirs and a team monitored compliance to existing guidelines on shipments and slaughter of FMD-susceptible animals. Three phases for eradication were used: (1) Control Phase (1996–2000) to reduce the incidence in high-risk areas and eliminating cases in remaining low-risk areas; (2) Consolidation Phase (2000–2004) with elimination of cases in the high-risk areas and intensification of disease monitoring and surveillance activities; and (3) Eradication Phase (2004–2009) with a significant reduction of outbreaks and implementation of the progressive zoning approach in Luzon (see Fig. 3) to achieve disease eradication and to serologically map Luzon to locate the last foci of infection [63, 64]. Strategic mass vaccination was applied in identified high-risk areas aimed at provision of a protective titre for FMD-susceptible animals, although post-vaccination monitoring raised doubts on vaccine effectiveness. Initially, to combat the pig-adapted CHY topotype, O_1_ Manisa vaccines produced in Europe were used. Later a homologous vaccine was used based on a pig-adapted local isolate O/Philippines/97.

In May 2001, the island of Mindanao received the status of FMD-free without vaccination followed shortly by the island groups of Visayas, Palawan and Masbate in May 2002 subsequent to serological surveys [63, 64]. The last reported outbreak of FMD in the Philippines occurred in December 2005 in Lukban, Quezon province in central Luzon [63, 64]. In November 2009, the Bureau of Animal Industry submitted documentation to support a status of ‘FMD-free without vaccination’ in northern and southern Luzon, and FMD-free with vaccination in central Luzon [63, 64]. Achieving these statuses would classify the Philippines as an ‘FMD-free country where vaccination is practiced’ under the guidelines provided by Article 8.5.3 of the 2009 OIE Terrestrial Animal Health Code [65]. Following the absence of FMD outbreaks for more than 3 years, a complete withdrawal of FMD vaccination was implemented, and the Philippines obtained the OIE recognition as FMD-free country without vaccination in June 2011 [64].

### Bilateral and multilateral co-operation FMD control programmes

Over the past 30 years, a number of bilateral and multilateral projects have provided assistance for the diagnosis and control of FMD in the SEA region as reviewed elsewhere [2, 66]. Projects have been managed and coordinated by the OIE, the Australian Centre for International Agricultural Research (ACIAR), the Australian Aid programme (formerly known as the Australian Agency for International Development (AusAid) and AIDAB), the European Union, the Government of South Korea and The Japan Trust Fund. The documented studies have been shown to be of benefit, although not without unique and significant technical and political challenges [2, 52]. The longest running of these projects is the SEA Foot and Mouth Disease Campaign (SEAFMD) [45, 67], involving the coordinated control of FMD by eight countries in the ASEAN region including Cambodia, Indonesia, Lao PDR, Malaysia, Myanmar, the Philippines, Thailand and Vietnam. The campaign is coordinated through a Regional Coordination Unit of OIE in Bangkok, with the support over the years from the Australian, Swiss and New Zealand Governments. In 2010, with Brunei Darussalam and Singapore as FMD-free countries, and the People's Republic of China (PR China) joining the campaign, it was renamed the Southeast Asia and PR China FMD campaign (SEACFMD). The programme was largely funded by the Australian Government through the Department of Foreign Affairs and Trade (DFAT) since 2011 under the ‘Stop Transboundary Animal Disease and Zoonoses’ (STANDZ) initiative [68], although recently, there has also been funding for SEACFMD from the Government of New Zealand. As of 2016, Mongolia is also an official member of SEACFMD, bringing the total number of member countries to 12 [69].

The SEACFMD 2020 Roadmap was initially developed with the aim of achieving FMD freedom with vaccination for SEA and PR China by the Year 2020 [45, 67]. It provided a long-term strategy that applies a progressive zoning approach to control FMD in the region and is a coordinated mix of policies and actions, involving progressive zoning, surveillance, emergency planning, vaccine supply, improved diagnostic capacity, traceability, training and community awareness. However, with increasing complexity of managing FMD in the region and evidence of introduction of new lineages of different serotypes in recent years indicating failures in achieving effective biosecurity, the Roadmap objective has been revised to achieve control rather than eradication by 2020. The lessons learned from past FMD control programmes and the research studies of current FMD events inform current knowledge and provide arguments for continued funding of FMD control programmes. Significant investments in the SEA region are required to improve technical capabilities in the field and improve control tools including vaccines, surveillance, biosecurity awareness and emergency disease response capabilities. Lapses in support for these initiatives will have serious impact on achieving the eventual global eradication of FMD.

## Discussion

FMD remains a major animal health problem in SEA with the current informal unregulated transboundary livestock trade necessitating that regional coordination of disease control activities, as occurs via the SEACFMD programme, be maintained. Lessons learned from both successful and unsuccessful FMD control programmes and recent and current research studies are critical for informing the future direction of control and eradication strategies.

### Animal and product movement biosecurity

Improved rigour in animal and product movement controls and biosecurity awareness together with adequate use of vaccination and public awareness proved effective in eradication of FMD in Indonesia and the Philippines, albeit with the advantages of island geography and infection by a single serotype. However, control of animal movement in the rest of the countries in SEA that share land borders is significantly more difficult. Outbreaks involving new virus lineages such as O/ME-SA/Ind-2001 that was likely introduced to the region, most likely from movement of infected animals or products from South Asia, provide evidence of increasing risk of FMD outbreaks in the region. Improved understanding of the dynamics of market chains, animal movement pathways and up-to-date disease status are critical to design and implement appropriate control programmes. Countries should also be employing animal identification schemes to assist in documenting movements. Further research is required that includes vaccine-related disciplines, improved diagnostics, economics, epidemiology and particularly implementation strategies that improve biosecurity awareness and practises. These initiatives could be complimented by risk-based approaches to the allocation of resources, along with improved real-time sharing of disease information to inform activities and improve coordination with major stakeholders.

### Serotypes, topotypes and diagnostics

An important issue is the large number (63.4%) of unserotyped FMD outbreaks in SEA, suggesting: (1) no samples or inappropriate samples were collected and submitted to a laboratory for serotyping purposes; (2) the quality of the sample submitted to the laboratory was poor (such as healing lesions, small size of sample or broken cold chain during storage and transport) with insufficient virus to enable a serotype diagnosis; (3) diagnostic assays or reagents were unable to detect the serotypes that are causing disease in SEA due to quality issues; or (4) the emergence of a novel serotype or topotype sufficiently genetically and antigenically different for diagnostic reagents to fail detecting the virus. Efforts are required to urgently address this issue or at least to define the reason(s) for this anomaly. To immediately address the issue of the novel serotype or topotype, sequencing of the VP1 gene of outbreak samples should occur early in an epidemic as a routine procedure and a review of diagnostic methodologies to enable regional standardisation should be performed.

### Vaccination

The field performance of an FMD vaccine depends on several factors including maintenance of cold chains, selection of the appropriate vaccine virus strains, correct vaccine administration procedures, efficient vaccination programme strategies and sufficient quantities of vaccine to achieve appropriate herd immunity levels to adequately suppress virus transmission. Field vaccine efficacy and effectiveness of vaccine administration should be closely monitored to ensure success of vaccination programmes. The true cost of vaccination programmes (e.g. production, cold chain, labour, delivery, training, etc.) should be estimated and sufficiently funded as well as monitored as achieving ‘coverage’ targets for effective FMD suppression in mass vaccination programmes in SEA has recently been shown to be challenging [70, 71]. FMD eradication programmes in the region should also include public awareness campaigns to encourage local engagement in implementing biosecurity measures and promoting the benefits of vaccination and other disease control measures (e.g. animal identification and movement control). However, achieving effective biosecurity education whilst undertaking field vaccination in SEA has also been shown to be challenging and it is recommended that these interventions be conducted separately and be part of training that has an objective of improving farmer incomes [70, 71]. Local commitment to improving biosecurity will improve the long-term sustainability of disease control and eradication programmes for FMD and the numerous other diseases that are likely to emerge from the increasingly dynamic trade in livestock and their products in the region and beyond.

To predict how well a vaccine will protect against a challenge virus of another strain within the same serotype requires vaccine-matching studies, involving *in vivo* cross-protection studies and harmonisation of *in vitro r*-value determinations between vaccine strains and field isolates [34]. There is an urgent need to prove the utility of contemporary vaccine strains in controlling the spread of field viruses causing FMD outbreaks, as the majority of published vaccine-matching studies from SEA are now only of historical value being from the 1980s and 1990s [21, 37, 38, 72].

### Factors contributing to the successful FMD eradication programmes

This review enables reflection on the reasons for success of the FMD eradication programmes in the Philippines and Indonesia. These eradication programmes in both countries had common themes. First, both are island archipelagos where limiting the spread of disease is simpler than on a continent with land borders between countries which enables easier reintroduction of existing or new viral serotypes/topotypes. Second, as both programmes were mostly only dealing with a single serotype (O) making decisions on formulation of vaccines uncomplicated. For the majority of continental SEA, multiple serotypes/topotypes must be considered in vaccine formulation. Another factor of importance was the use of staged phases of the eradication such as in the Philippines with the Bicol surveillance buffer zone. To achieve a similar result on continental SEA would require significant political will and cooperation, technical expertise, human resources to achieve compliance with controls and a large dose of luck to enable eradication. The Bicol surveillance buffer zone was also used to monitor the performance of vaccines using post-vaccination serology, a very important component of any successful FMD vaccination programme. ‘Stamping out’ may have a role in the latter stages of a campaign and was employed although in a very limited manner primarily in Indonesia and even less in the Philippines where it was used as a public awareness tool. However, it is very unlikely that this would be culturally or economically appropriate for many countries in SEA where smallholder livestock production systems predominate.

It is important to note that both of these eradication programmes were supported by the Australian government which committed significant long-term funding and technical assistance to ensure the success of the programmes. Ensuring continuity of funding and provision of technical expertise is important in any disease eradication programme and significant economic and human resources will be required to achieve this FMD eradication on the SEA continent.

Further, application of locally derived research outcomes is necessary, particularly in understanding the priorities, motivations and drivers of smallholder farmers and other stakeholders at risk of FMD and of animal health authorities leading disease control programmes. A change management approach has been advocated to encourage a more systematic delivery and appraisal of FMD control and eradication programmes in SEA [71]. This follows extensive research where smallholder farmers in parts of Lao PDR and Cambodia were enrolled in large-scale multiyear projects and trained in preventive animal health, animal nutrition, forage development, animal marketing and reproduction. A biosecurity change management framework was developed to understand: (1) motivation for change, (2) resistance to change, (3) knowledge management, (4) cultural dimensions, (5) farming systems priorities and (6) leadership. Improved communication of applied biosecurity awareness and practices for FMD control in smallholder communities is now being recognised as a key requirement for scaling up FMD risk mitigation. Although commercial livestock operations are developing in SEA, the majority of livestock owners are likely to remain as smallholders for a considerable period of time and to encourage their participation in disease control, inclusion of non-biosecurity initiatives targeting improved productivity and smallholder farmer livelihoods is considered necessary [71].

Lessons may also be learned from other successful FMD control programmes such as those progressing in South America [73, 74], although as these involve mostly commercial operations there are major differences between these FMD zones. Factors contributing to the success of the South American programme include coordination with the private sector and other institutions, coordination of national and subnational programmes, training of human resources, consolidation of official veterinary services and awareness of the important role of livestock producers and the private sector in the implementation and financing of vaccination and other control actions [74]. Factors that negatively influenced the South American FMD control programme include weak structure of official veterinary services, insufficient political commitment, use of international trade as the main driving force rather than farmer livelihoods, managerial weakness, lack of explicit monitoring and evaluation processes, and not fully understanding the epidemiology and risks associated with the spread of FMD [74].

### International cooperation

Challenges facing the programme include unregulated animal movements; difficulties of vaccine application and its efficacy; a low-level of field technical capacity; insufficient biosecurity; low levels of local engagement in disease control and lack of emergency disease response capacity; and difficulties in co-ordination of national and international control programmes. Continued funding of FMD control programmes is required, particularly to improve technical capabilities in the field and laboratory, and improve the implementation of disease control tools, including vaccines, surveillance, biosecurity, compliance with movement controls and public awareness programmes for FMD control. While there is an improved knowledge of the market situation of domestic animals and their products at inter-district, inter-provincial or even international level [5, 6] including trade with PR China, there is a continued need to better understand the livestock market systems within Asia and their influence on the spread of FMD.

## Conclusion

FMD control in SEA is unlikely to be achievable without a true regional commitment to collaboration. Challenges facing the programme include unregulated animal movements; difficulties of vaccine application and its efficacy; a low-level field technical capacity; insufficient biosecurity practices; low levels of local engagement of smallholders in disease control and lack of emergency disease response capacity; and difficulties in co-ordination of national and international control programmes. Continued funding of FMD control programmes is required, particularly to improve technical capabilities in the field and improve the implementation of disease control tools, including vaccines, surveillance, biosecurity, compliance with movement controls and public awareness programmes for FMD control. However, FMD control and freedom will be difficult to achieve in the short to medium term as each country has its own national disease control and resource allocation priorities and modernisation of livestock production systems is slow. For example, one country will have its own policy about live animal imports or frozen meat which may be counter-aligned to those of others in the trading bloc. The Chinese government is likely to have an important role to play in FMD stewardship, although this may be complicated by PR China not being an ASEAN member state. FMD control and freedom is a step-by-step process that requires continued integration of effort and approach, especially in facilitating trade, managing risk and implementing biosecurity. If regional/transnational trade systems become more formalised, then it may be possible to reduce the current risks from ‘informal’ markets in FMD transmission. All of these factors are key to the control and freedom from FMD in the foreseeable future.
